# Endoscopic Transsphenoidal Cisternostomy for Nonneoplastic Sellar Cysts

**DOI:** 10.1155/2015/389474

**Published:** 2015-01-22

**Authors:** Yukai Su, Yudo Ishii, Chien-Min Lin, Shigeyuki Tahara, Akira Teramoto, Akio Morita

**Affiliations:** ^1^Comprehensive Cancer Center of Taipei Medical University and Department of Neurosurgery, Taipei Medical University-Shuang Ho Hospital, Ministry of Health and Welfare, New Taipei City, Taiwan; ^2^Department of Neurosurgery, Teikyo University Chiba Medical Center, Chiba, Japan; ^3^Department of Neurosurgery, Taipei Medical University-Shuang Ho Hospital, Ministry of Health and Welfare, New Taipei City, Taiwan; ^4^Department of Neurological Surgery, Graduate School of Medicine, Nippon Medical School, Tokyo 113-8603, Japan; ^5^Japan Labour Health and Welfare Organization, Tokyo Rosai Hospital, Tokyo, Japan

## Abstract

*Background and Importance*. Sellar arachnoid cysts and Rathke's cleft cysts are benign lesions that produce similar symptoms, including optochiasmatic compression, pituitary dysfunction, and headache. Studies have reported the use of various surgical treatment methods for treating these symptoms, preventing recurrence, and minimizing operative complications. However, the postoperative cerebrospinal fluid (CSF) fistula and recurrence rate remain significant. *Clinical Presentation*. In this paper, we present 8 consecutive cases involving arachnoid cysts and Rathke's cleft cysts, which were managed by using drainage and cisternostomy, the intentional fenestration of the cyst into the subarachnoid space, and then meticulously closing sellar floor using dural sutures. The postoperative images, CSF fistula rate, and the recurrence rate were favorable. *Conclusion*. We report this technique and discuss the benefit of this minimally invasive approach.

## 1. Introduction

The sellar nonneoplastic cystic lesions include Rathke's cleft cysts and arachnoid cysts [1, 2]. The operation targets to them are symptomatic relief and avoidance of complication. The more the cyst wall the surgeon removes, the more the risk of recurrence decreases, but the more the risk of hypothalamic injury and pituitary dysfunction increases. And communication with subarachnoid space (SAS) causes the risk of cerebral spinal fluid (CSF) fistula. In order to prevent CSF fistula, the surgeon leaves more cyst wall and avoids communication with subarachnoid space, but the recurrence rate increases. The recurrence and cerebral spinal fluid (CSF) fistula rate remains not ignorable, despite whether microscopically transsphenoidal approach or endoscopically transsphenoidal surgery [3–7]. The surgical management for these cysts has been a challenge.

In this paper, we present 8 cases of Rathke's cleft cysts and arachnoid cysts, managed with endoscopically transsphenoidal surgery by applying intentional fenestration to the subarachnoid space and closing the sellar floor using delicate dural suturing technique. This method is minimally invasive and the surgical results were favorable.

## 2. Methods

### 2.1. Patient Population and Data Collection

All patients were included in the prospective database between October 2009 and August 2013 who underwent endoscopic endonasal transsphenoidal surgery for treating symptomatic Rathke's cleft cysts and arachnoid cysts. The sample of 8 patients comprised 2 males and 6 females, with ages ranging from 37 to 73 years. In all of the subsequent patients, no packing of fat or other grafts was performed, wide fenestration of the cyst cavity to the SAS was intentionally conducted, and the sellar dura was closed meticulously using sutures and fibrin glue.

Patient clinical notes, operative notes, imaging studies, and hormonal studies were reviewed. In addition, data on lesion characteristics, detailed intraoperative observations, intra- and postoperative complications, and clinical outcomes were collected. A single surgeon, Yudo Ishii, performed all of the procedures.

### 2.2. Preoperative and Postoperative Evaluations

#### 2.2.1. Endocrine Assessment

Pituitary function was assessed using standard hormonal assays, including the levels of thyroid-stimulating hormones (TSHs) and thyroxine (Free-T3, Free-T4), growth hormones (GHs) and IGF-I, plasma adrenocorticotropic hormones (ACTHs) and serum cortisol, prolactin, luteinizing hormones (LHs), follicular-stimulating hormones (FSHs), and testosterone in men. In the early postoperative period, the patients were monitored for DI based on urine volume and urine-specific gravity. The hormone levels of the patients were monitored every 2 days after the operation.

#### 2.2.2. Visual Function Assessment

The preoperative and postoperative visual function assessment involved measuring visual acuity using formal visual field testing. Visual function was considered improved if the visual acuity assessed using the handheld eye card improved by at least 2 lines or if the visual field defects, assessed using field confrontation or by having an ophthalmologist conducting a formal visual field test review, were resolved or improved.

#### 2.2.3. Imaging

All of the patients underwent pre- and postoperative pituitary MR imaging with and without Gadolinium enhancement, including early postoperative MR imaging on day 7 and subsequently within 3–6 months after operation. One of the female patients, the sixth patient, received a preoperative MR exam, but during the preoperative evaluation, a cardiac pacemaker was prescribed and, therefore, the postoperative image study was replaced with a brain CT scan.

### 2.3. Surgical Techniques

A direct endoscopic endonasal transsphenoidal surgery (ETSS) was performed in all of the cases in this study. The surgical procedure used for treating the RCCs and ACs in this study is summarized briefly, as follows.

**Figure 1:**
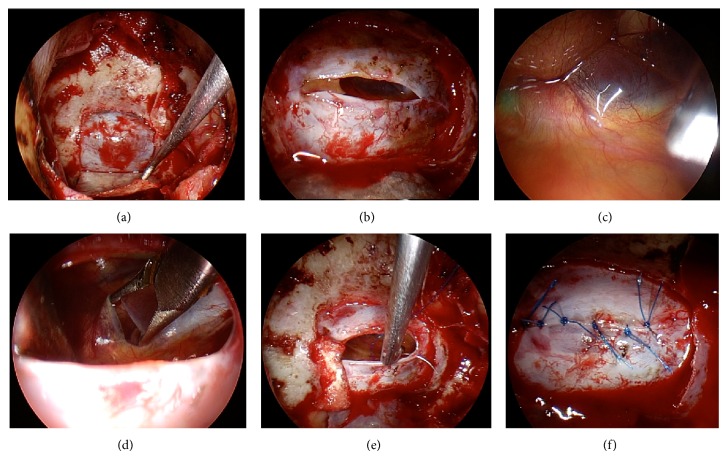


**Figure 2:**
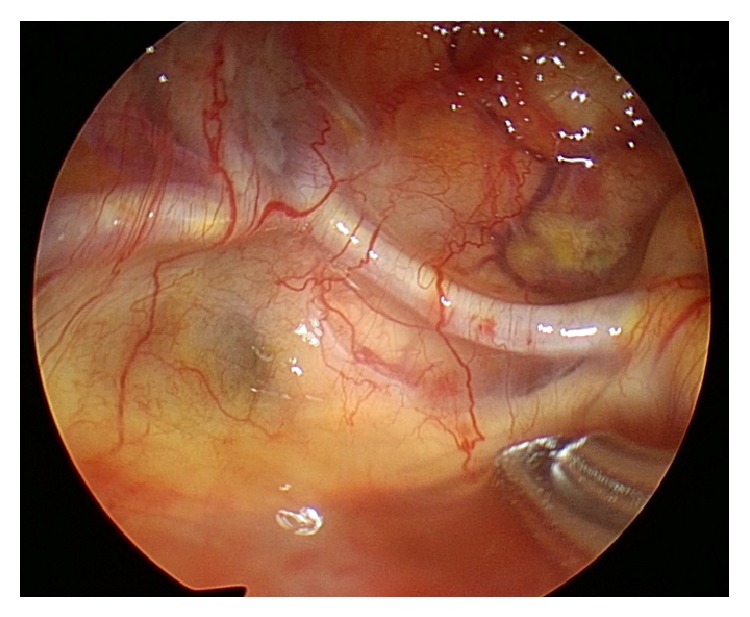


**Figure 3:**
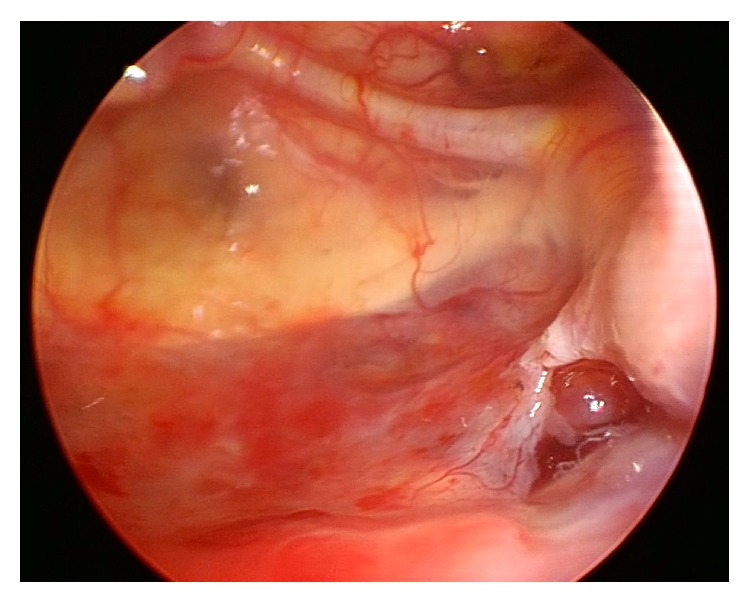


After performing a wide sphenoidotomy and sellar floor opening (Figure 1(a)), the dura was incised horizontally (Figure 1(b)). The content of the RCCs was removed using suction and irrigation. The cyst wall was then inspected by inserting an endoscope at 0°, 30°, and 70° angles. In addition, the subarachnoid membrane, pituitary stalk, and the dorsum sellae were identified (Figure 1(c)). The bilateral anterior communicating arteries and optic chiasm were also clearly observed (Figure 2). The cyst wall was biopsied and sent for pathology exam. Fenestration of the subarachnoid space in the cystic wall was performed using bipolar coagulation and sharp scissors (Figure 1(d)). This step was performed carefully to avoid injuring the basilar artery behind the arachnoid membrane. The posterior communicating artery was identified after the fenestration (Figure 3). The cyst wall was not removed from the pituitary gland because of the risk of worsening the pituitary function. After communication with the subarachnoid space, the dura was closed with interrupted sutures using 6-0 nylon and the easy slipknot technique (Figures 1(e) and 1(f)) [8]. No fat or other grafts were used for packing the cyst cavity. The sellar floor was reconstructed using an autologous sellar bone, a nasal septum bone, or artificial absorbable plate. Fibrin glue was then applied to the surgical field and the nostril was packed with gauze. No nasal septal flap, lumbar drain, or acetazolamide was used.

## 3. Results

### 3.1. Patient Demographic Data (Table 1)

Three arachnoid cyst and 5 Rathke's cleft cyst cases were included in this series. All of the patient symptoms were visual disturbances. The patients did not exhibit headache or pituitary dysfunction, except for the fifth patient who had a headache, and the headache dissipated after the operation. The visual function of all of the patients was improved after the surgery. The preoperative MR image and the postoperation 3-4-month image are shown in Table 2. No CSF fistula developed among these 8 patients, and the visual disturbances subsided in all of the patients. Case number 8 with recurring Rathke's cleft cyst received previous microscopically transsphenoidal surgery 5 years ago and had visual disturbance, and the symptom resolved after the endoscopic transsphenoidal drainage with cisternostomy. In this case,* Staphylococcus epidermidis* was cultured from the cyst content. He did not experience fever or meningitis postoperatively. But the patient received oral antibiotics for 4 weeks. The postoperative image in 3 months showed shrinkage of RCC. The RCC recurred in 6 months postoperatively. He received the same procedure with more thorough cyst content removal and fenestration into the subarachnoid space. The dura closure was achieved with suturing of his fascia lata.  Table 3 is the postoperation result of case number 8.

## 4. Discussion

An arachnoid cyst is a collection of CSF-like fluid, the walls of which comprise an arachnoid structure. The cyst can develop at any site in the subarachnoid space along the cerebrospinal axis. Two theories on the pathogenesis of intrasellar arachnoid cysts have been postulated [3]. The first theory was proposed by Benedetti et al. [13], who stated that intrasellar arachnoid cysts are initially formed by a largely communicating subarachnoid space expanding into the sella turcica. Blocking the communication between the suprasellar subarachnoid space and the cyst through a meningitic, hemorrhagic, or inflammatory event then isolates the arachnoid cyst. The second theory is that a large diaphragmatic aperture combined with a pulsatile CSF force allows the suprasellar subarachnoid space to penetrate the sella turcica. The pituitary stalk and gland participate in a ball-valve mechanism, which reoccludes the dural defect after the CSF enters [3]. The symptoms of intrasellar arachnoid cysts might be related to the internal pressure that causes the adjacent structure to be compressed and blood flow circulation impaired, resulting in headaches, visual function defects, and pituitary dysfunction. To relieve these symptoms, the pressure inside the cyst should be normalized.

Intended fenestration of the cystic wall balances the pressure between the outside and inside of the cyst and can subsequently relieve the symptoms. The other way is that one of the pathogeneses of arachnoid cyst is the ball-valve mechanism. Once fenestration of the cyst with the subarachnoid space was performed, no pressure gradient occurred and, thus, the recurrence of arachnoid cyst was less likely. The wider the fenestration is, the faster the communication of subarachnoid space with the cyst is. The size of the fenestration was variable, depending on the relation between the small perforating arteries and the pituitary gland. We usually make fenestration until the flow of CSF becomes the to-and-fro oscillations of the arachnoid membrane like the 3rd ventriculostomy.

**Figure 4:**
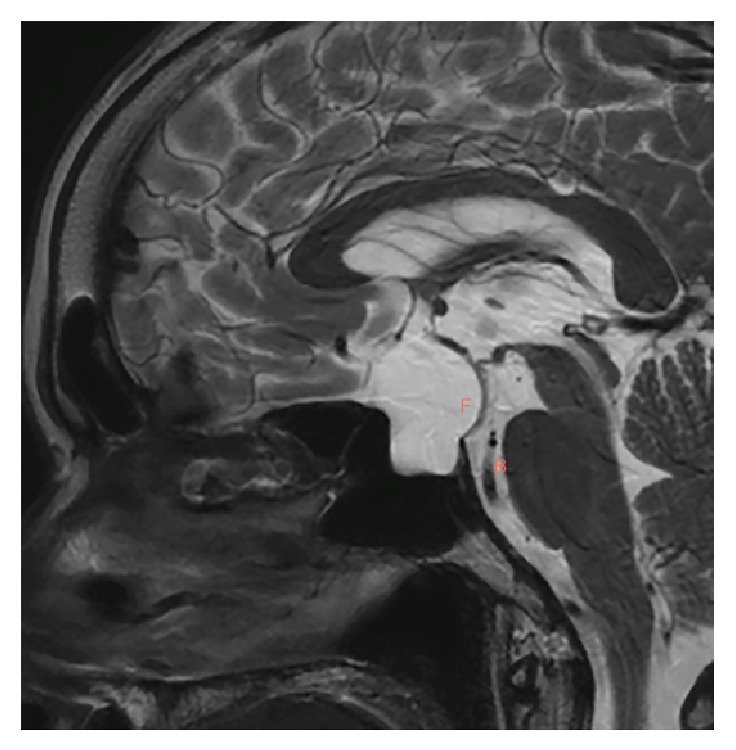


We recommend that the fenestration site be located at the arachnoid membrane of the dorsum sellae, just above the posterior clinoid process. For Rathke's cleft cyst, the anterior pituitary gland is mostly located at the ventral sella, and the posterior lobe is at the dorsal sella. In endoscopic transsphenoidal surgery, we could verify the location of normal pituitary gland and the perforators supplying it. We make fenestration-avoiding injury to these vessels by direct vision under endoscope. In case of arachnoid cysts, pituitary gland would be located in the dorsal or caudal sella. In such cases, fenestration would be made at the frontal part of cyst as described by Oyama et al. Before the surgery, the surgeon should carefully examine the sagittal view of the MR image of the brain to determine the relationship of the trunk and tip of the basilar artery behind the dorsum sellae and possible location of pituitary stalk (Figure 4).

Surgical therapy is the most commonly used method for treating symptomatic primary and recurrent Rathke's cleft cysts, and transsphenoidal approach is the preferred approach [10]. Several neurosurgeons have advocated performing total cyst wall resection to reduce the recurrence rate, but complete Rathke's cleft cyst wall resection is associated with high rates of postoperative DI and pituitary dysfunction [9, 10, 14, 15].

Consequently, most neurosurgical centers limit the use of this complete resection method [11]. The goal of performing a cystic wall biopsy is to determine the pathology of the lesion and simple drainage of the cyst to release the cystic pressure that causes symptoms to occur. These goals are similar for treating both arachnoid cysts and Rathke's cleft cysts. Thus, we reviewed management between these 2 types of diseases to subsequently analyze the treatment results and prognosis.

In the treatment of Rathke's cleft cysts, fenestration of the cyst cavity to the subarachnoid space after removing the cyst content relieved the cyst pressure. The symptoms may thus be remedied. However, because the cyst wall was not completely removed, some residual ciliated epithelial cells may form the cyst content. The epithelial cell might be with a mucus secreting function, and the communication of subarachnoid space may prevent this secretion from accumulating. In the literature, studies have reported that “chemical meningitis” had occurred when removing the cystic craniopharyngioma [16]. None of studies in the literature reported the occurrence of postoperative chemical meningitis after craniotomy of Rathke's cleft cysts, even in suprasellar Rathke's cases involving partial cyst wall removal [17, 18]. Oyama et al. published the method of transsphenoidal cyst cisternostomy with a keyhole dural opening [12]. The microscopical approach and the extended transsphenoidal approach were used. The CSF leakage was prevented by dural plasty using the fascia lata and stitching with 6-0 monofilament sutures. Their result showed visual symptoms improved and none of the patients required reoperation for postoperative CSF leakage. Therefore, we propose that the fenestration of the Rathke's cleft cyst wall to the subarachnoid space may reduce the risk of cyst recurrence.

The postoperative recurrence rate and complication rate are high for these 2 diseases. The literature review and our result were summarized (Table 4).

CSF fistula is a major postoperative complication of endoscopic transsphenoidal surgery, especially in cystic lesions [2]. Fat, gelfoam, and collagen sponge packing, bony sellar floor reconstruction using fibrin glue, and nasal septal flap reconstruction have become common methods for CSF fistulas in endoscopic transsphenoidal surgery. However, these sella and sphenoid sinus-packing materials are also the source of occult infection. For RCCs, the risk of recurrence may be related to the rate of infection [19].

Therefore, we recommend not packing the cyst with fat or other materials after fenestration of the RCC with communication of the SAS. Although the flow of CSF may be large, the dura of the sellar floor can be closed using meticulously applied sutures and the synergistic easy slipknot approach, as reported by the senior researcher [8].

We performed operations, totally 151 Rathke's cleft cysts and 5 arachnoid cysts from 2004 to 2013 in Nippon Medical School University Hospital with the method of traditional drainage and cyst wall biopsy. Among them, 10 Rathke's cleft cysts and 2 arachnoid cysts recurred. All the recurrent cases were large cysts with suprasellar extension. We propose the indication of this fenestration procedure for the large cysts with suprasellar extension. This approach would not be too invasive if it is performed in hands of an experienced endoscopic transsphenoidal surgeon with good sellar reconstruction technique, like the dura suturing. Endoscopic transsphenoidal management of the cystic lesions could be as easy as in craniotomy cases.

## 5. Conclusion

Managing symptomatic RCC and sellar AC by fenestration of the cyst wall and meticulously applying dural sutures can provide symptom relief and prevent recurrence without increasing the risk of CSF fistula complications. Endoscopic endonasal transsphenoidal surgery to the cyst lesions can achieve more minimally invasive result than the extended approach method using microscope.

## Supplementary Material

The Supplementary Material is a demonstration video of this approach technique. This case is a recurrence of Rathke's cleft cyst. Endoscopically cyst drainage, cyst wall fenestration, dura suturing, and sellar reconstruction were demonstrated.

## Figures and Tables

**Table 1:** Patient demographics, clinical data in 8 cases of AC and RCC.

Patient number	Age (yr)/sex	Diagnosis	Maximum diameter (mm)	Headache	Visual disturbance	Pituitary/hypothalamus disfunction
1	45/F	RCC	23.15	Nil	Positive	Nil
2	64/F	AC	51.88	Nil	Positive	Nil
3	71/F	RCC	22.54	Nil	Positive	Nil
4	37/M	AC	37.01	Nil	Positive	Nil
5	53/F	AC	26.68	Positive	Positive	Nil
6	56/M	RCC	28.02	Nil	Positive	Nil
7	73/F	RCC	25.48	Nil	Positive	Nil
8	59/F	RCC recurrence	23.45	Nil	Positive	Nil
		Previous with fat packing				

AC: arachnoid cyst, RCC: Rathke's cleft cyst.

**Table 2:** 

Patient number	Preoperative image	Postoperative 3-month image
(1) (RCC)	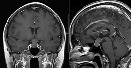	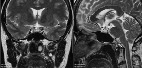

(2) (AC)	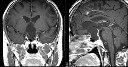	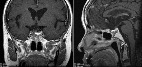

(3) (RCC)	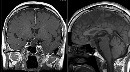	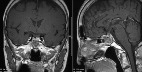

(4) (AC)	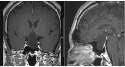	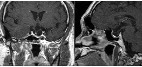

(5) (AC)	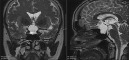	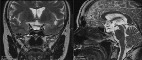

(6) (RCC)	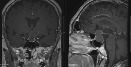	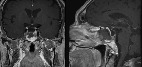

(7) (RCC)	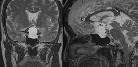	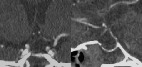

**Table 3:** Preoperative image and postoperative image in recurrence RCC patient.

Patient number	Preoperative image	Postoperative image
(8) (RCC recurrence)	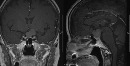	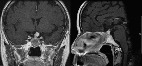

(8) (Recurrence after fenestration)	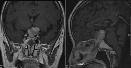	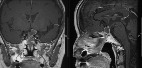

**Table 4:** 

AC or RCC	Author and year	Case numbers	Decompression method	Packing or reconstruction method	Complications	Recurrence
AC	Dubuisson et al. (2007) [3]	9	Microscopically, cyst removed totally (2) and partially (7), communicating with SAS	Adipose tissue (4/9), bone pieces, biological glue, lumbar puncture drainage	1 permanent diabetes insipidus (11%); 2 CSF fistula (22%)	FU from 2 months to 324 months, 0 recurrence

AC + RCC	Cavallo (2008) [2]	AC: 10 RCC: 20	AC: microscopic or endoscopic, no cyst wall removal; RCC: endoscopic (20), cyst removed totally in purely suprasellar lesion, partially in sellar lesion	AC: adipose tissue and/or collagen sponge; RCC: 7 with reconstruction, 13 left open	AC: 2 CSF fistula (20%); RCC: 1 thalamic infarction (5%), 2 diabetes insipidus (10%), 1 CSF fistula (5%)	AC: FU 10 to 94 months, 1 recurrence (10%); RCC: FU 7 to 70 months, 2 recurrence (10%)

AC	Mclaughlin et al. (2012) [6]	8	Microscopically or endoscopic approach, no cyst wall removal	Adipose tissue, titanium micromesh, fat and collagen buttress, acetazolamide for 48 hours	No	FU 6 to 47 months, 2 recurrence (25%)

RCC	Benveniste et al. (2004) [9]	62	Microscopically sublabial (37), endonasal (23), endoscopic endonasal (1) craniotomy (1), cyst wall removed totally (6)	Adipose tissue (19) + bone piece (55) or titanium mesh (1); left open (6)	1 CSF fistula (1.6%), 1 abdominal fat graft harvest infection	FU 1 to 166 months, 10 recurrence (16%)

RCC	Aho et al. (2005) [10]	118	Microscopically sublabial (118), 114 cyst wall removed totally,	Adipose tissue (43)	22 diabetes insipidus (19%), 1 CSF fistula (0.8%), 1 meningitis (0.8%)	FU over 60 months, 21 recurrence (18%)

RCC	Lillehei et al. (2011) [11]	82	Microscopically sublabial and endonasal, simple cyst drainage, alcohol cauterization	Gelfoam and bone strut, fibrin glue, spinal drain for intraoperative CSF leakage, 0 adipose tissue packing	2 CSF fistula (2.4%), 3 transient DI (3.7%)	FU 4 to 163 months, 8 recurrence (9.7%)

RCC	Park et al. (2012) [7]	73	Microscopically and endoscopic assisted, cyst drainage	34 packing adipose tissue, 22 packing surgically, 17 no packing, sellar reconstruction with bone, porous polyethylene, TachoComb with BioGlue	2 CSF fistula (2.7%)	FU 12–166 months, 12 recurrence (16%)

AC + RCC	Oyama et al. (2014) [12]	AC: 6; RCC: 1	Microscopically extended approach, cisternostomy	7 dura stitches, no fat packing	1 CSF fistula	FU 36 to 49 months, 2 recurrence (28%)

AC + RCC	Our series	AC: 3; RCC: 5	Endoscopically endonasal, cyst drainage cisternostomy	8 dura stitches, no fat packing, bone and BioGlue	0 CSF fistula	FU 4 to 50 months, 1 recurrence (12%)
